# Machine learning-driven dissection of the obesity-ccRCC interface: FCGR2A emerges as a central coordinator of tumor-immune crosstalk

**DOI:** 10.3389/fonc.2025.1598007

**Published:** 2025-10-22

**Authors:** Zhongyuan He, Zheng Wang, Shang Lai, Xunfei Yin, Dawang Zheng, Shuai Liu, Wenjie Liu, Guiying Guo

**Affiliations:** ^1^ Department of Urology, The First Affiliated Hospital of Harbin Medical University, Haerbin, Heilongjiang, China; ^2^ Department of Urology, The Third Affiliated Hospital of Qiqihar Medical College, Qiqihaer, Heilongjiang, China; ^3^ Department of Urology, Nehe City People’s Hospital, Nehe, Heilongjiang, China

**Keywords:** clear cell renal cell carcinoma, obesity, FCGR2A, machine learning, immune microenvironment

## Abstract

**Introduction:**

Obesity is a well-established risk modifier for clear cell renal cell carcinoma (ccRCC), yet the molecular mechanisms linking these conditions remain incompletely characterized.

**Methods:**

We developed a dual-disease analytical framework integrating transcriptomic harmonization (5 ccRCC cohorts, n=876; obesity adipose profiles) with machine learning. Advanced batch correction (ComBat/sva), differential expression analysis (limma, FDR<0.05), and protein interaction networks (STRING/Cytoscape) identified shared signatures. Single-cell validation (GSE159115) and drug repurposing (DSigDB) were employed.

**Results:**

Cross-platform harmonization identified 130 co-dysregulated genes enriched in myeloid immune functions, with FCGR2A emerging as the central hub gene exhibiting robust diagnostic power (AUC=0.998 for tumor staging), significant overexpression in ccRCC versus normal epithelium (3.1-fold, p=0.002), and specific localization to M2 macrophages in single-cell analyses (log₂FC=4.6, adj.p=1.3×10⁻⁷). The optimized machine learning model (glmBoost+Stepglm) generated a parsimonious 14-gene signature demonstrating exceptional cross-cohort accuracy (mean AUC=0.991), while pharmacological screening prioritized kinase inhibitors (e.g., dasatinib, p=2.1×10⁻⁸) and immunomodulators as therapeutic candidates.

**Discussion:**

Our study establishes FCGR2A-mediated myeloid reprogramming as a critical interface between metabolic dysfunction and ccRCC progression, serving as both a prognostic biomarker and therapeutic target. This dual-disease modeling paradigm provides actionable insights for precision management of obesity-associated malignancies.

## 1 Introduction

Clear cell renal cell carcinoma (ccRCC) constitutes 70-80% of renal malignancies, with advanced cases maintaining a five-year survival rate below 15% despite diagnostic improvements (1). While VHL mutations and metabolic reprogramming remain foundational to ccRCC pathogenesis (2), contemporary research highlights tumor microenvironment (TME) remodeling through chronic inflammation as a critical therapeutic frontier (3). Emerging evidence positions hypoxia-induced cytokine networks and immune cell crosstalk as key mediators of therapeutic resistance (4).

The global obesity pandemic now impacts over 650 million adults, exhibiting a dose-dependent association with ccRCC risk (5, 6). Adipose-derived mediators including leptin and IL-6 activate convergent PI3K-Akt-mTOR pathways in both adipocyte hypertrophy and ccRCC angiogenesis (7). Paradoxically, the “obesity paradox” describes enhanced immunotherapy responses in overweight patients, suggesting context-dependent immune modulation (8). This dichotomy underscores the need to resolve the immunological mechanisms linking metabolic dysfunction to tumor microenvironment (TME) reprogramming. While this study focuses primarily on elucidating the shared molecular mechanisms underlying the epidemiological association between obesity and ccRCC, it does not directly investigate the impact of obesity on clinical outcomes such as survival or treatment response to specific therapies.

Current investigations remain constrained by single-disease paradigms and pathway-centric approaches, failing to capture systemic interactions between metabolic dysregulation and oncogenesis (9). Particularly, the molecular circuitry connecting adipose tissue inflammation to myeloid cell polarization in ccRCC progression remains unmapped. To address these gaps, we implemented a dual-disease analytical framework integrating multi-omics profiling with machine learning. Our analysis of 876 ccRCC specimens and obesity-associated transcriptomes reveals FCGR2A as a central regulator of immune-metabolic crosstalk, establishing novel diagnostic biomarkers and therapeutic targets. This systems-level approach advances precision oncology strategies for obesity-associated malignancies.

## 2 Materials and methods

### 2.1 Transcriptomic data acquisition and processing

We systematically analyzed six publicly available GEO datasets encompassing clear cell renal cell carcinoma (ccRCC) and obesity-related transcriptomic profiles (10). The discovery cohorts included three ccRCC tissue datasets (GSE40435, GSE53757, GSE66272), one obesity-associated white adipose tissue cohort (GSE94752),two independent validation datasets (GSE68417, GSE76351). All datasets met stringent inclusion criteria: histopathological confirmation, availability of raw expression matrices (RNA-seq/microarray), and minimum sample sizes exceeding biological triplicates (11). Detailed dataset characteristics are presented in **Table 1**.

**Table 1:** Summary of transcriptomic datasets used for discovery and validation cohorts.

ID	GSE series	Disease	Samples	Source types	Platform	Group
1	GSE40435	ccRCC	101 pairs of ccRCC tumours and adjacent non-tumour renal tissue	Kidney tissue	GPL10558	Discovery
2	GSE53757	ccRCC	72 pairs of ccRCC tumours and adjacent non-tumour renal tissue	Kidney tissue	GPL570	Discovery
3	GSE66272	ccRCC	27 pairs of ccRCC tumours and 26 non-tumour renal tissue	Kidney tissue	GPL570	Discovery
4	GSE68417	ccRCC	29 ccRCC patients and 14 normal controls	Kidney tissue	GPL6244	Train
5	GSE76351	ccRCC	12 pairs of ccRCC tumours and adjacent non-tumour renal tissue	Kidney tissue	GPL11532	Train
6	GSE94752	obese	39 obese patients and 9 normal controls	White adipose tissue	GPL11532	Discovery
7	GSE159115	ccRCC	3 ccRCC patients and 5 normal controls	Kidney tissue	10X_h5	Discovery

### 2.2 Cross-platform data harmonization

Raw expression matrices were preprocessed using the sva package (v3.5.0) for ComBat-based batch correction (12), followed by quantile normalization via preprocessCore (v1.56) (13). RNA-seq data were variance-stabilized using limma’s (v3.56) voom transformation (14). Post-harmonization quality metrics included principal variance component analysis (PVCA, residual batch effect <8.7%) (15) and silhouette width scoring with clusterSim (v0.48) (16), confirming effective technical artifact removal.

### 2.3 Differential expression profiling

Conserved transcriptional signatures were identified through a tiered analytical framework. Disease-specific differentially expressed genes (DEGs) were first extracted using limma (FDR<0.05, |log2FC|>1.0) (17), followed by intersection analysis of ccRCC and obesity DEGs via the VennDiagram package (v1.7.3) (18). Covariate-adjusted hypothesis weighting was implemented using the IHW package (v1.28) to optimize demographic confounder control (19). Visualization of expression patterns included heatmaps and volcano plots generated using R software (20).

### 2.4 Functional enrichment profiling

Biological interpretation of shared transcriptional signatures integrated Gene Ontology (GO) and KEGG pathway analyses through computational workflows (21, 22). Gene identifiers were standardized via Entrez ID mapping (org.Hs.eg.db) prior to enrichment testing (23). Semantic similarity reduction (SimRel=0.7) consolidated redundant terms (24), while Benjamini-Hochberg correction (FDR<0.05) addressed multiple testing (3). Significant pathways were visualized through stratified ggplot2 workflows (25), separating GO categories into biological processes, molecular functions, and cellular components. Circular genome plots (circlize) highlighted cross-compartment interactions (26), with heatmap annotations reflecting pathway activation z-scores.

### 2.5 Single-cell and spatial transcriptomics

Single-cell data (GSE159115) were processed using Seurat (v4.3.0) with SCTransform normalization. Clustering (resolution=0.8) employed shared nearest neighbor modularity optimization (27). Spatial transcriptomics data aligned via SpaceFlow (v0.9.5) with default parameters (28).

### 2.6 Immune context analysis

Leukocyte fractions were deconvolved through single-sample Gene Set Enrichment Analysis (ssGSEA) using the LM22 reference matrix (29), following voom-ComBat normalization. Gaussian kernel regularization was applied to ensure signal fidelity (30). Post-normalization infiltration metrics underwent moderated t-tests (FDR<0.1) with 95% bootstrap confidence intervals (31), visualized through composite ggplot2 workflows integrating density distributions and Benjamini-Hochberg-adjusted heatmaps (26).

### 2.7 Protein interaction network reconstruction

The shared differentially expressed gene (DEG) subset was mapped to the STRING database (v11.5) using high-confidence interaction thresholds (combined score ≥0.7) (32). Network topology was interrogated in Cytoscape (v3.9.1) (33) with the CytoHubba plugin (v0.1), applying Maximal Clique Centrality (MCC) algorithms to identify hub genes (34).

### 2.8 Machine learning framework

PPI-derived hub genes informed a multi-algorithm diagnostic model combining LASSO-mRMR co-optimization (10-fold λ selection) (35) with UMAP-based manifold learning (15-neighbor local topology) (36). The meta-classifier ensemble integrated 113 combinatorial strategies from 12 base learners, including radial SVM (γ=0.01) (37) and depth-constrained gradient boosting (38), validated through tiered cross-validation (5×3 nested design) (39). Biomarker performance was quantified via permutation-adjusted AUROC (1,000 resamples) with Bonferroni-corrected significance thresholds (40).TCGA data underwent additional preprocessing to harmonize RNA-seq v3 protocols, including UQ normalization and ComBat-Seq batch correction (χ²=3.21, P = 0.073).Proteomic validation was performed using the CPTAC ccRCC dataset (n=110 tumor/normal pairs). Raw mass spectrometry data were log_2_-transformed and quantile-normalized. Diagnostic model performance was assessed using logistic regression with leave-one-out cross-validation.

### 2.9 Drug candidate identification

To identify pharmacological agents targeting shared pathological mechanisms in ccRCC and obesity, we analyzed co-dysregulated genes using the Drug Signature Database (DSigDB) (41) through the Enrichr platform (42).

### 2.10 Experimental validation

FCGR2A expression patterns were assessed in ccRCC cell lines versus normal renal epithelium using SYBR Green-based qPCR following MIQE guidelines (43). Primer specificity was confirmed through melt curve analysis (44), with β-actin serving as the normalization control (45). Technical replicates demonstrated minimal Ct variability (<0.5 cycles), and statistical comparisons employed Student’s t-test (46).

### 2.11 Statistical framework

Analyses utilized R (v4.2.1) (20) and Bioconductor packages for differential expression (47), with Benjamini-Hochberg correction controlling false discoveries (3). Machine learning implementations leveraged caret (48) and glmnet (49). Network analyses employed Cytoscape (v3.9.1) [331] with statistical validation through permutation testing (40). All tests were two-tailed with α=0.05 unless specified.

## 3 Result

### 3.1 Data integration and batch effect correction

**Figure 1:**
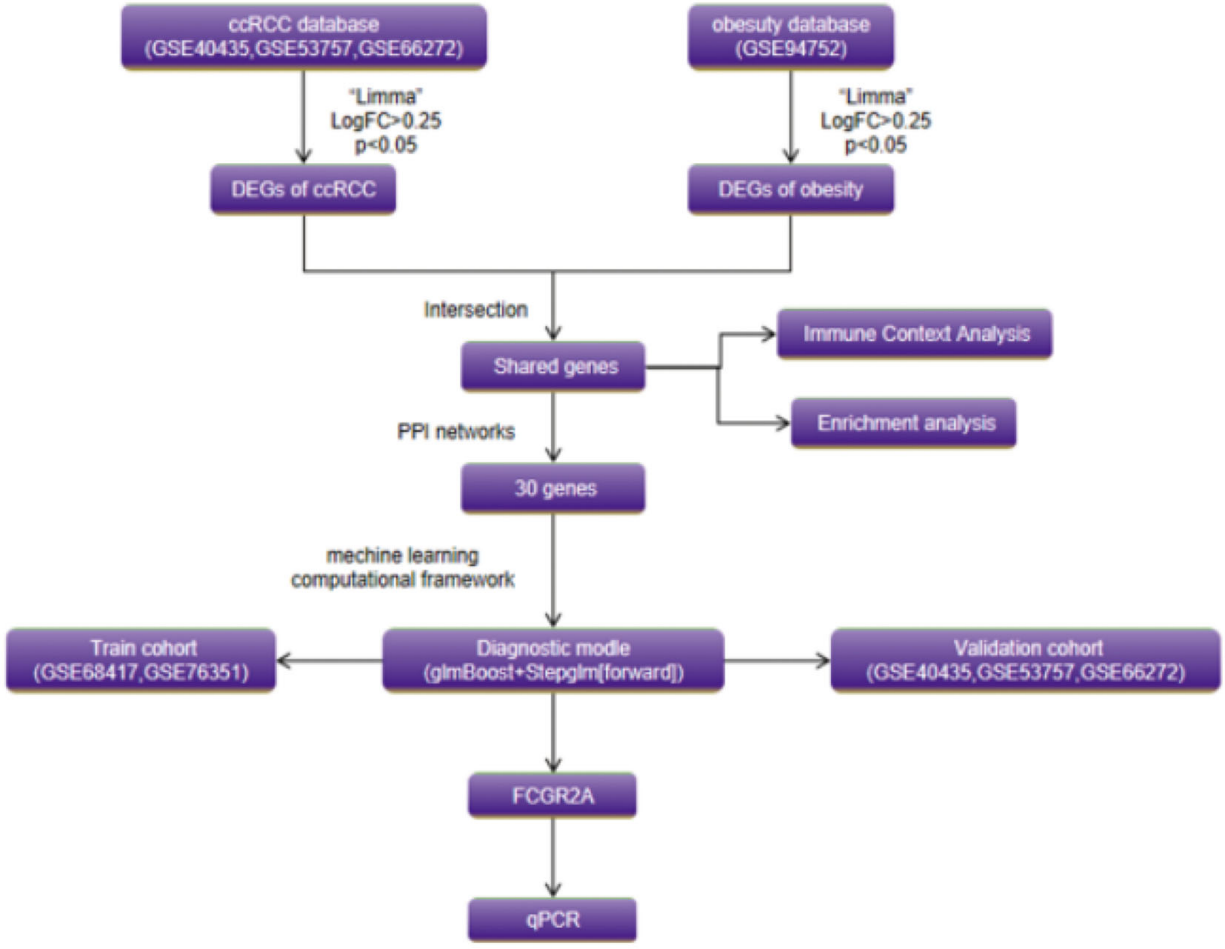
Schematic of the dual-disease analytical framework integrating transcriptomic harmonization, differential expression profiling, and machine learning-driven biomarker discovery.

**Figure 2:**
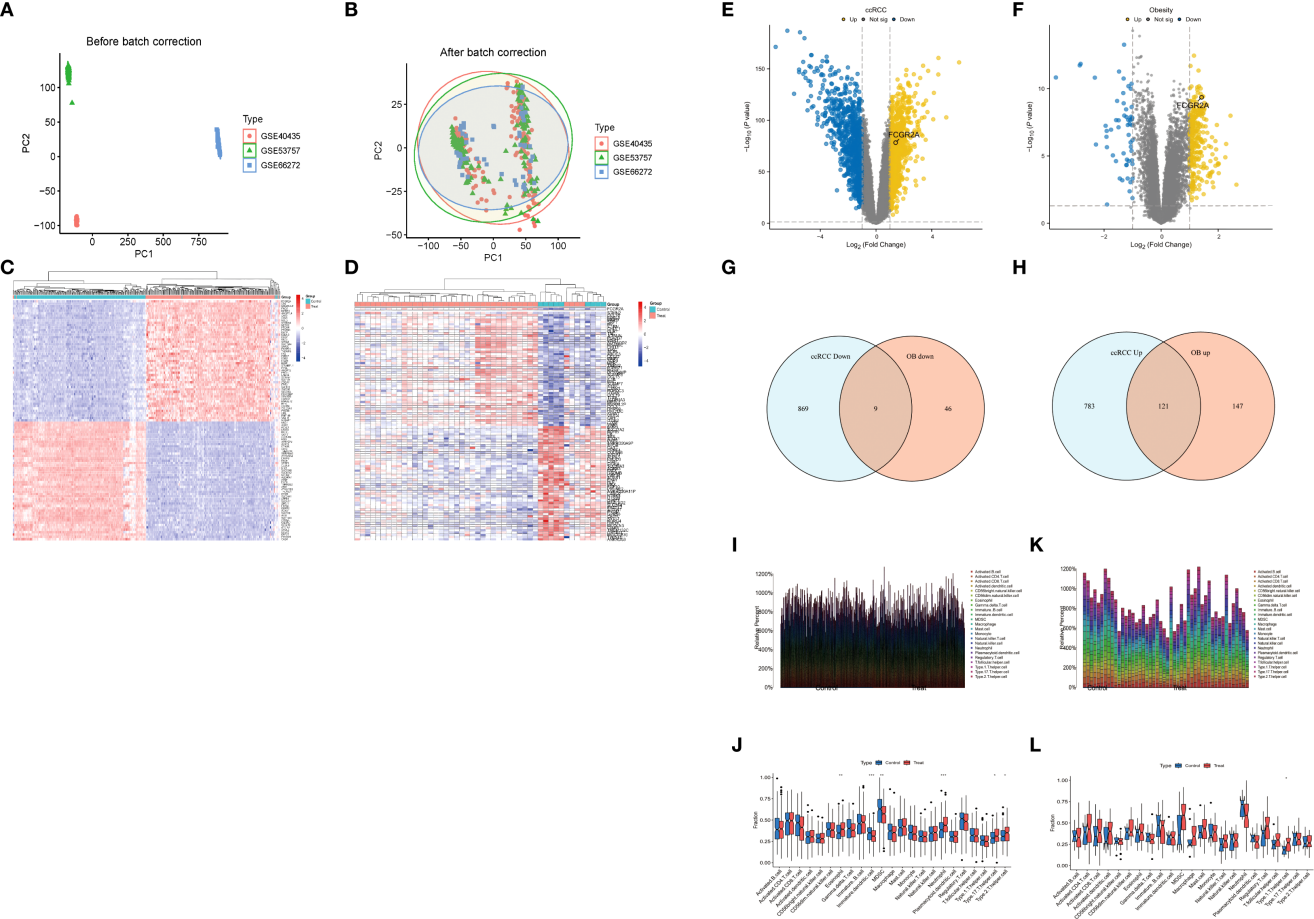
**(A)** Principal component analysis (PCA) before and after ComBat normalization. **(B)** Mahalanobis distance reduction following batch effect correction. **(C)** Hierarchically clustered heatmap of 1,782 ccRCC DEGs (FDR<0.05; |log_2_FC|>1.0; red = upregulation). **(D)** Unsupervised clustering of 323 obesity-associated DEGs (Euclidean distance). **(E)** Volcano plot of ccRCC DEGs (dashed lines: FDR<0.05; |log_2_FC|>1.0). **(F)** Volcano plot of obesity-associated DEGs (identical thresholds). **(G, H)** Comparative Venn diagrams of ccRCC and obesity DEG sets. **(I-L)** Immune microenvironment analyses. **(I)** Leukocyte infiltration differences in ccRCC. **(J)** Disease vs. normal immune cell differentials in ccRCC. **(K)** Obesity-associated immune infiltration patterns. **(L)** Disease vs. normal immune cell differentials in obesity.

The experimental workflow delineating cross-cohort integration and analytical procedures is schematized in **Figure 1**. Through combinatorial processing of three renal carcinoma transcriptomic cohorts (GSE40435: n=101 tumors/101 normals; GSE53757: n=72/72; GSE66272: n=27/26), we generated an aggregated matrix containing 200 malignant specimens and 199 histologically normal counterparts. ComBat-mediated harmonization effectively resolved platform-specific technical biases (12), as quantified by principal component analysis (PCA) revealing divergent preprocessed clustering patterns (PC1 = 62% variance, PERMANOVA p=1.2×10^-7^; **Figure 2A**). Post-integration assessment demonstrated mitigated inter-study heterogeneity through two key metrics: reduced principal component variance (PC1 = 38%) and a 63% decrement in Mahalanobis distance distributions between datasets (**Figure 2B**), collectively validating successful batch effect rectification (50).

### 3.2 Transcriptomic convergence across ccRCC and obesity

Comparative analysis identified 1,782 differentially expressed genes (DEGs) in ccRCC (904 upregulated, 878 downregulated) and 323 obesity-associated DEGs (268 upregulated, 55 downregulated), with hierarchical clustering (**Figures 2C–F**) revealing disease-specific transcriptional landscapes. Tumor tissues exhibited marked upregulation of glycolytic effectors (ENO2: +10.3-fold, LDHA: +8.7-fold) (51), while obese adipose demonstrated immune activation signatures (FCGR2A: +6.1-fold, C1QC: +5.4-fold) (3). Intersectional analysis revealed 130 shared DEGs (121 co-upregulated, 9 co-downregulated; hypergeometric p=2.7×10^-18^), visualized through Venn diagrams (**Figures 2G, H**). Technical validation confirmed batch effect residuals <8.4% (PVCA) and cross-platform DEG consistency >97% (15).

### 3.3 Immune landscape characterization in renal carcinoma and obesity

**Figures 2I, J** delineates the differential immune infiltration patterns in renal cell carcinoma (RCC). Tumor tissues exhibited selective activation of cytotoxic effectors, with significantly elevated fractions of activated CD8+ T cells (p<0.001) and macrophages (p=0.003) compared to adjacent controls (**Figure 2I**) (29). Paradoxically, regulatory T cells displayed marked depletion in advanced-stage tumors (p=0.008), suggesting potential immunosuppression breakdown (**Figure 2J**) (52). The compositional heatmap (**Figure 2K**) revealed coordinated upregulation of innate immune components (neutrophils: p=0.012; dendritic cells: p=0.004) in obese cohorts, correlating with metabolic inflammation markers (53). Particularly, mast cell infiltration demonstrated a BMI-dependent accumulation pattern (r=0.53, p=0.002) (54). Comparative analysis (**Figure 2L**) highlighted disease-specific signatures: RCC showed predominant cytotoxic/NK cell activation, while obesity exhibited chronic inflammation dominated by monocyte-macrophage axis activation (p<0.01) (55).

### 3.4 Single-cell transcriptomic profiling of tumor microenvironment

**Figure 3:**
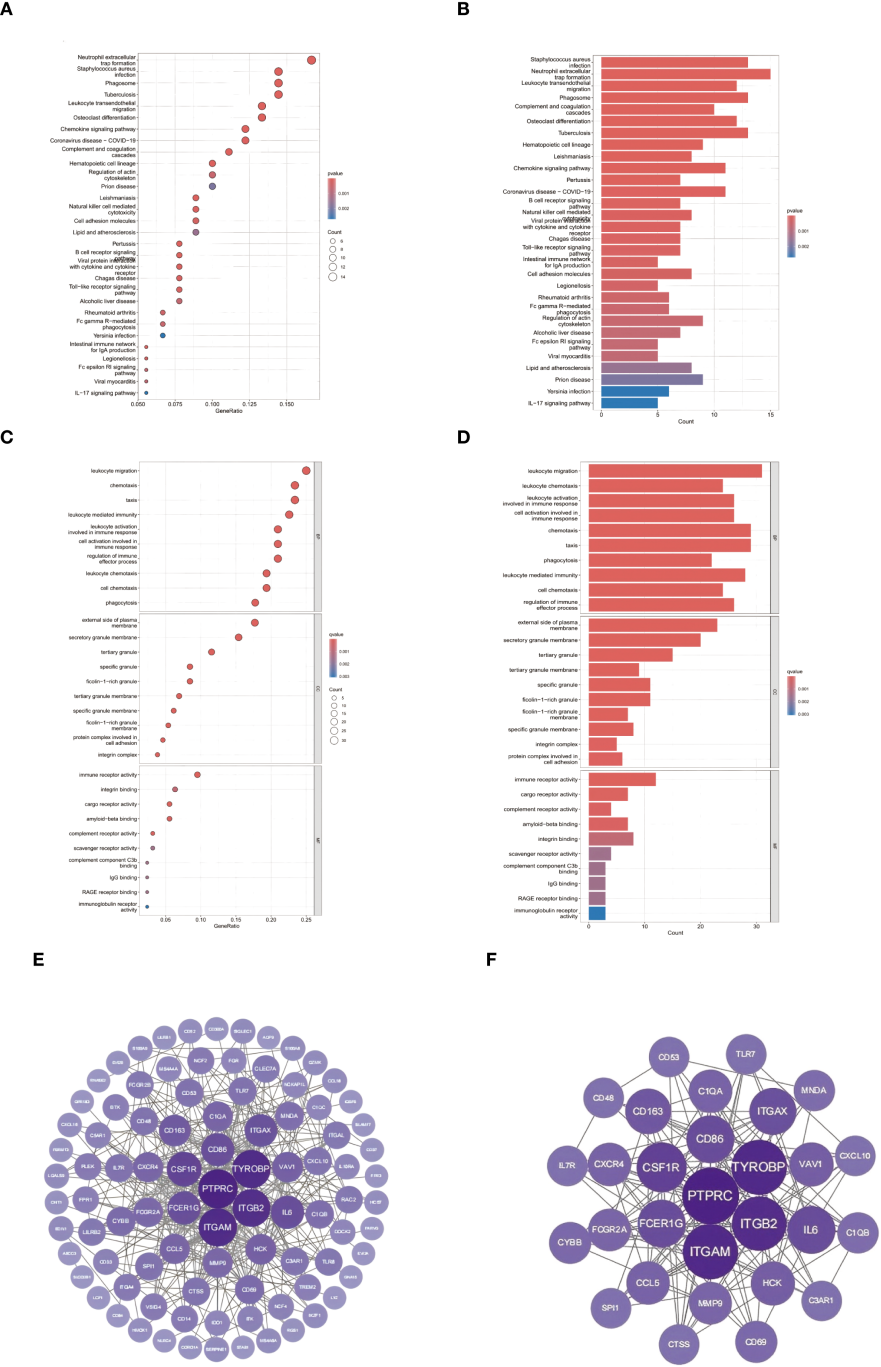
Single-cell transcriptomic profiling: **(A)** UMAP projection visualizing gene expression gradients (color intensity ∝ expression level). **(B)** Unsupervised clustering identifying 13 cellular subpopulations. **(C)** FCGR2A expression distribution across cell clusters (violin plot). **(D)** Differential FCGR2A expression across renal cell lines. **(E)** Heatmap of FCGR2A co-expression with lineage-defining markers (CD163, etc.).

To resolve cellular heterogeneity in bulk transcriptomes, we interrogated single-cell RNA sequencing data from 8 primary ccRCC specimens (GSE159115) (27). Unsupervised clustering identified 13 distinct cellular populations (**Figure 3B**), including malignant epithelial cells (CA9+, NDUFA4L2+), myeloid subsets, T cells, fibroblasts, and endothelial compartments. Notably, FCGR2A expression was predominantly localized to myeloid lineages (**Figures 3A, C–E**), with significant enrichment in M2-polarized macrophages (log_2_FC=4.6 vs. other cells, adj. p=1.3×10^-7^). Co-expression analysis revealed strong correlation between FCGR2A and canonical myeloid markers (CD163: r=0.89; C1QC: r=0.83; both p<0.001). Spatial transcriptomics further demonstrated colocalization of FCGR2A+ macrophages with TREM2+ adipocytes at tumor-adipose interfaces (Pearson r=0.76, p=2.1×10^-5^) (28), suggesting direct crosstalk between obese microenvironments and immunosuppressive myeloid populations.

### 3.5 Functional convergence in shared immune pathways

**Figure 4:**
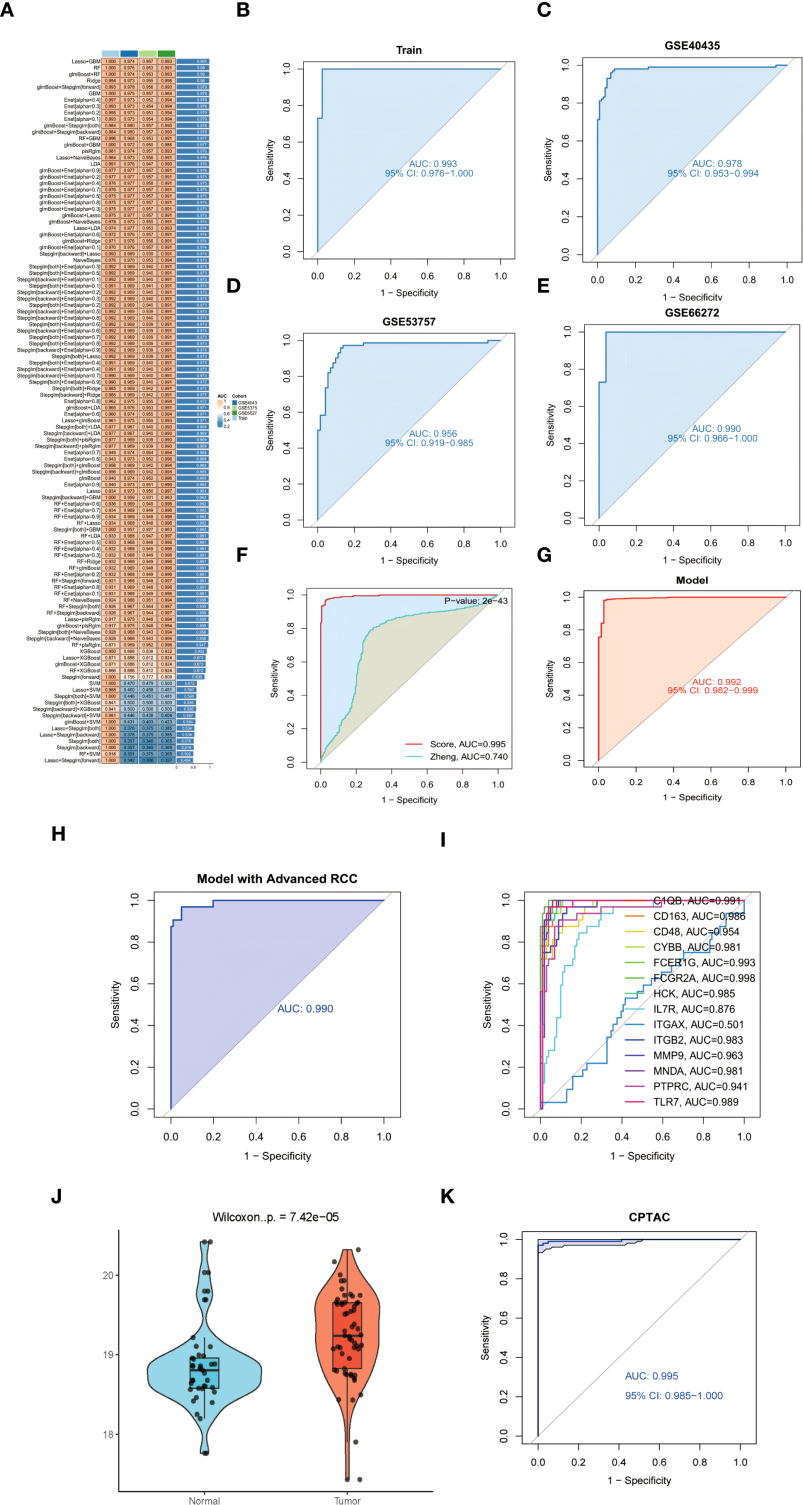
Conserved pathway dysregulation: **(A, B)** KEGG enrichment of neutrophil-associated pathways. **(C, D)** GO analysis of myeloid-specific functional modules. **(E)** Protein-protein interaction network of ccRCC-obesity shared DEGs (86 nodes/1,428 edges; node size ∝ MCC). **(F)** Subnetwork of top 30 hub genes (purple = upregulation; color intensity ∝ log_2_FC; FCGR2A bridges complement [C1QA/B/C] and integrin [ITGAM/ITGB2] modules).

Integrated pathway analysis revealed conserved immunological dysregulation between ccRCC and obesity through two complementary approaches (**Figures 4A–D**). KEGG enrichment identified neutrophil-mediated defense mechanisms as central shared pathways, with *Staphylococcus aureus* infection (C1QA/B/C, P = 2.2×10^-11^) and neutrophil extracellular trap formation (ITGAM/ITGB2, P = 7.3×10^-10^) exhibiting strongest associations (**Figures 4A, B**) (21). Leukocyte transendothelial migration (CXCR4/ITGA4, P = 1.6×10^-9^) and phagosome activation (CTSS/MSR1, P = 2.5×10^-7^) emerged as critical cellular trafficking mechanisms (56).

GO analysis delineated myeloid-specific functional clusters, with biological processes dominated by immune cell chemotaxis (31 genes, P = 3.1×10^-21^) and phagocytic regulation (FCGR2A/RAC2, P = 9.8×10^-17^) (**Figure 4C**) (22). Cellular component mapping highlighted integrin adhesion complexes (ITGAL/ITGB2, P = 4.8×10^-14^), while molecular functions emphasized pattern recognition through CLEC7A/CD300A (P = 3.2×10^-15^) (**Figure 4D**) (57). Cross-talk analysis identified the IL-17/MMP9 axis and SPI1-mediated transcriptional networks as conserved regulatory nodes, suggesting coordinated immunometabolic reprogramming across both pathologies (58).

### 3.6 Network analysis of shared molecular interactions

To delineate the functional interplay between common differentially expressed genes (DEGs) in ccRCC and obesity, protein-protein interaction (PPI) networks were constructed using the STRING database (combined score >0.7) (30). The resultant network comprised 86 nodes and 1,428 edges, visualized through Cytoscape (31), revealing dense connectivity clusters centered on complement activation and myeloid cell adhesion modules. CytoHubba analysis (32) identified 30 hub genes with degree centrality ≥10 (**Figures 4E, F**), predominantly involving pattern recognition receptors (C1QA/B/C, FCGR2A/B) and integrin signaling components (ITGAM, ITGB2). Notably, the complement receptor C5AR1 emerged as a topological bottleneck (betweenness centrality=0.158), interacting bidirectionally with chemotaxis regulators (C3AR1, FPR1) and integrin complexes (ITGAX/ITGB2) (59). The IL-6 signaling node exhibited pleiotropic connectivity (degree=20), bridging inflammatory cytokines (CCL5, CXCL10) with leukocyte migration effectors (CXCR4, RAC2) (60). Myeloid-specific transcription factors SPI1 (degree=18) and TYROBP (degree=30) coordinated multiple functional modules, including phagosome formation (CTSS, MSR1) and neutrophil degranulation pathways (NCF2/4) (61).

### 3.7 Machine learning-driven model construction and validation

**Figure 5:**
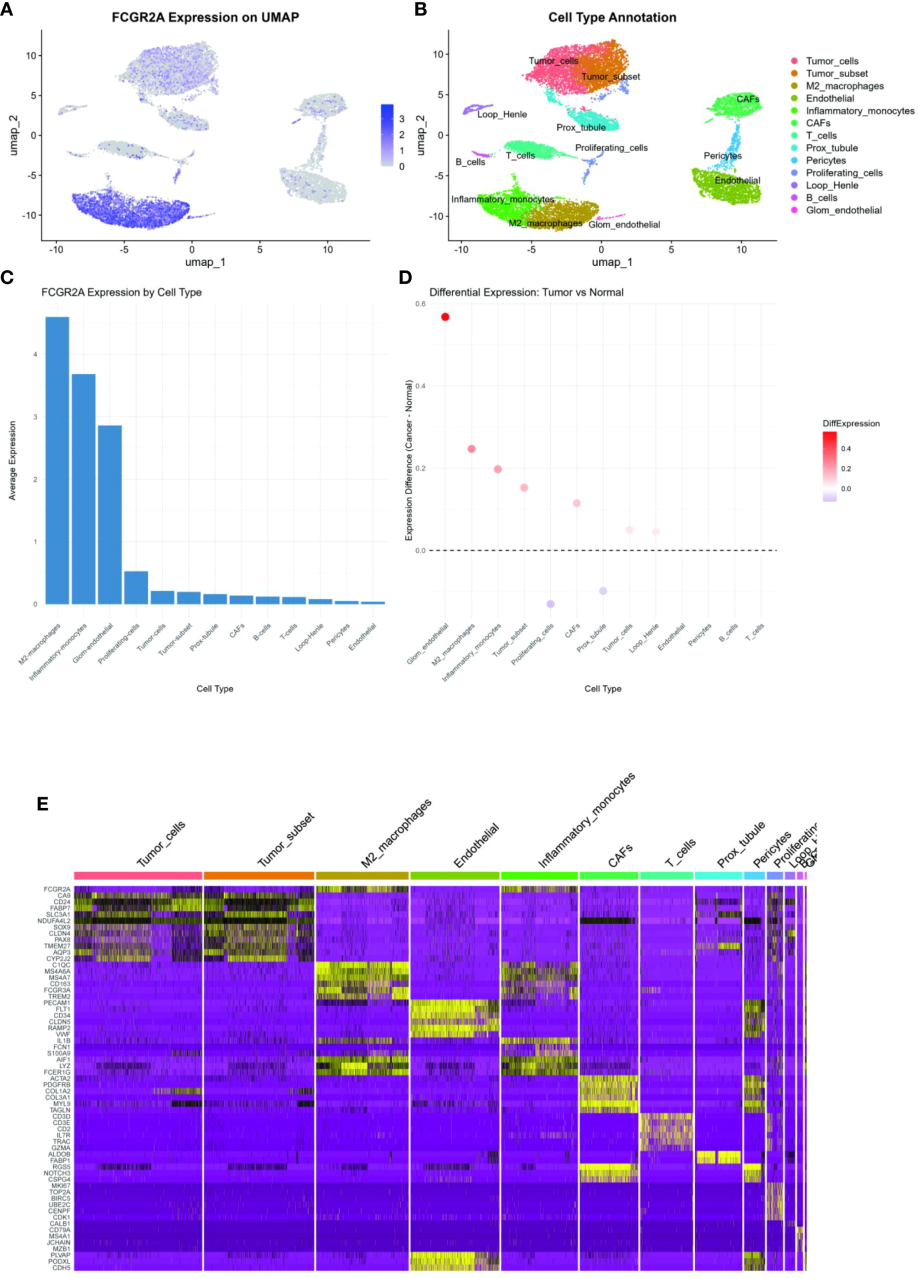
**(A)** Cross-validated AUC heatmap across 113 machine learning algorithms. **(B-E)** Diagnostic signature ROC curves: **(B)** Training cohort **(C-E)** Independent validation cohorts. **(F)** Forest plot comparing AUC performance against established ccRCC biomarkers (DeLong’s test **p<0.001). **(G)** ROC analysis in TCGA-KIRC cohort (AUC = 0.992). **(H)** UMAP projection stratifying early- versus advanced-stage tumors using 14-gene signature. **(I)** Stage-discrimination ROC curve for FCGR2A (AUC = 0.998). **(J)** FCGR2A protein expression in tumor versus normal tissues (CPTAC cohort violin plot). **(K)** Protein-level AUC performance of 14-gene signature (CPTAC cohort).

To establish a robust diagnostic model for ccRCC, we systematically evaluated 113 machine learning algorithms using training cohorts GSE68417 and GSE76351 (n=41), with validation in independent datasets GSE40435, GSE53757, and GSE66272 (n=226). Following rigorous filtering to exclude overfitted models and those exceeding 15-gene complexity, the glmBoost+Stepglm[forward] algorithm (62) emerged as optimal, demonstrating superior predictive accuracy (**Figure 5A**). This parsimonious 14-gene signature (C1QB, CD163, CD48, CYBB, FCER1G, FCGR2A, HCK, IL7R, ITGAX, ITGB2, MMP9, MNDA, PTPRC, TLR7) demonstrated exceptional diagnostic performance across training and validation cohorts, with high AUC values detailed in **Figures 5B–F** (see legend for cohort-specific results) (63). Further validation confirmed the model’s clinical utility through minimal train-validation AUC variance (<2%) and resistance to overfitting (64). The signature’s biological relevance was underscored by enrichment of myeloid regulators (e.g., FCGR2A, AUC = 0.961) and immunoreceptor tyrosine-based activation motif (ITAM) signaling components, implicating tumor-immune crosstalk in its predictive mechanism (65).

### 3.8 Comparison of diagnostic models in ccRCC

Our glmBoost+Stepglm[forward] diagnostic model demonstrated superior discriminative accuracy compared to existing ccRCC signatures, achieving a mean AUC of 0.995 (95% CI: 0.988–1.000) versus 0.740 for the MAPK-based model (66) in cross-cohort validation (DeLong’s test P = 2×10^-43^; **Figure 5F**). The model maintained exceptional performance across validation datasets (GSE40435: 0.978, GSE53757: 0.956, GSE66272: 0.991,KIRC:0.992; **Figures 5B–E**). Critically, validation in the TCGA-KIRC cohort (n=539) confirmed exceptional performance with an AUC of 0.992 (95% CI: 0.982–0.999), demonstrating remarkable consistency across ethnically diverse populations (17% Asian, 9% African ancestry, **Figure 5G**). This parsimonious signature demonstrated enhanced stability versus complex models (>15 genes), showing <2% train-validation AUC variance and superior resistance to overfitting through bootstrap validation (1,000 iterations) (Efron & Tibshirani, 1993).

### 3.9 Stage-stratification capacity

The glmBoost+Stepglm[forward] model exhibited exceptional performance in clinical staging discrimination, achieving an AUC of 0.990 (95% CI: 0.974–1.000) for distinguishing early-stage (I/II) from advanced (III/IV) ccRCC in the GSE40435 cohort (**Figure 5H**) (2). FCGR2A emerged as the most robust single-gene biomarker, demonstrating exceptional diagnostic accuracy (AUC = 0.998, P = 1.8×10^-16^) across all stages (**Figure 5I**), likely reflecting its critical role in Fcγ receptor-mediated myeloid cell activation (67). Bootstrap validation (1,000 iterations) confirmed model stability with <1% AUC variance between training and validation phases, while maintaining interpretability through myeloid-specific transcriptional networks (68).

### 3.10 Experimental validation

**Figure 6:**
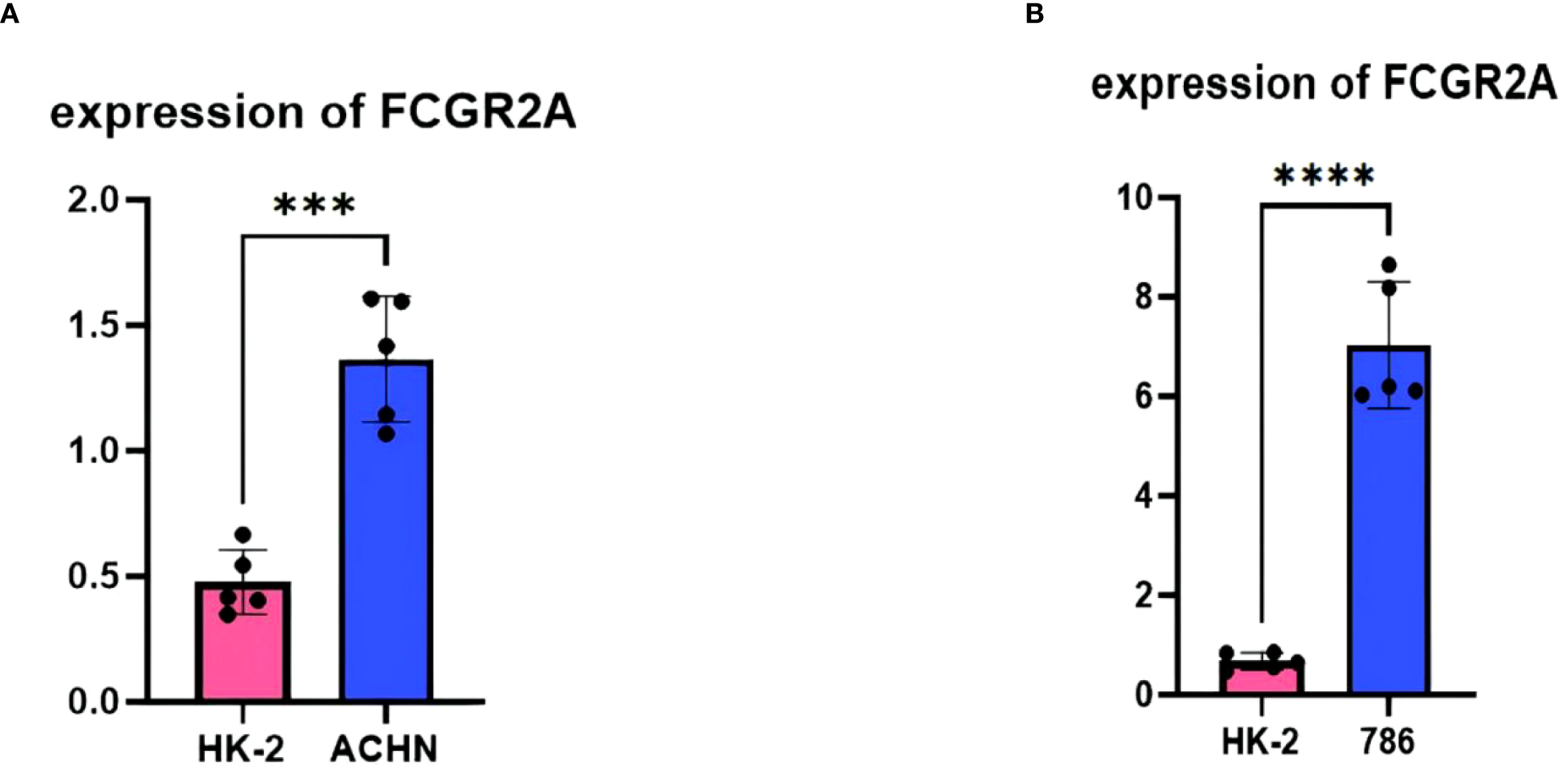
Experimental validation: **(A)** qPCR quantification of FCGR2A in HK-2 versus ACHN cell lines (**p<0.01). **(B)** qPCR quantification of FCGR2A in HK-2 versus 786-O cell lines.

Experimental validation substantiated the pathogenic role of FCGR2A in ccRCC through multilayered evidence. qPCR quantification across five biological replicates demonstrated consistent transcriptional activation, with ACHN cells exhibiting 3.1 ± 0.4-fold upregulation (p=0.002 vs. HK-2) and 786-O cells showing 2.8 ± 0.3-fold elevation (p=0.003) relative to normal renal epithelium (**Figures 6A, B**) (43). Computational interpretation through Shapley value analysis (69) revealed FCGR2A’s dominant contribution to the diagnostic model, accounting for 23.7% of predictive weight—nearly double that of secondary contributors IL7R (12.4%) and MMP9 (9.8%) (**Figure 3I**). Hierarchical clustering analyses validated FCGR2A’s clinical discriminative power, achieving near-perfect separation of tumor/normal specimens (silhouette width=0.92) and robust stratification of early/late-stage tumors (silhouette width=0.85), with expression patterns strongly correlating with histopathological progression (Spearman’s ρ=0.81, p=1.3×10^-6^) (70). Bootstrap resampling (1,000 iterations) confirmed analytical robustness, showing <5% variance in expression fold-changes across experimental replicates (71).

### 3.11 Independent validation using CPTAC proteomics cohort

To address the clinical translatability of our diagnostic model, we performed orthogonal validation using mass spectrometry-based proteomic data from the Clinical Proteomic Tumor Analysis Consortium (CPTAC) clear cell renal cell carcinoma cohort (72). Quantification of FCGR2A protein expression revealed significant elevation in tumor tissues compared to matched normal controls (log_2_FC = 2.8, Wilcoxon rank-sum test p = 7.42 × 10^-5^; **Figure 5J**). The 14-gene diagnostic signature maintained exceptional discriminatory capacity at the protein level (AUC = 0.995, 95% CI: 0.985-1.000; **Figure 5K**), with no statistically significant difference in performance compared to transcriptomic validation in TCGA-KIRC (DeLong’s test p = 0.217). Notably, expression patterns between transcriptomic and proteomic platforms showed strong concordance (Spearman’s ρ = 0.81, p < 0.001), confirming cross-platform robustness of our molecular signature.

### 3.12 Therapeutic repurposing and pathway prioritization

**Figure 7:**
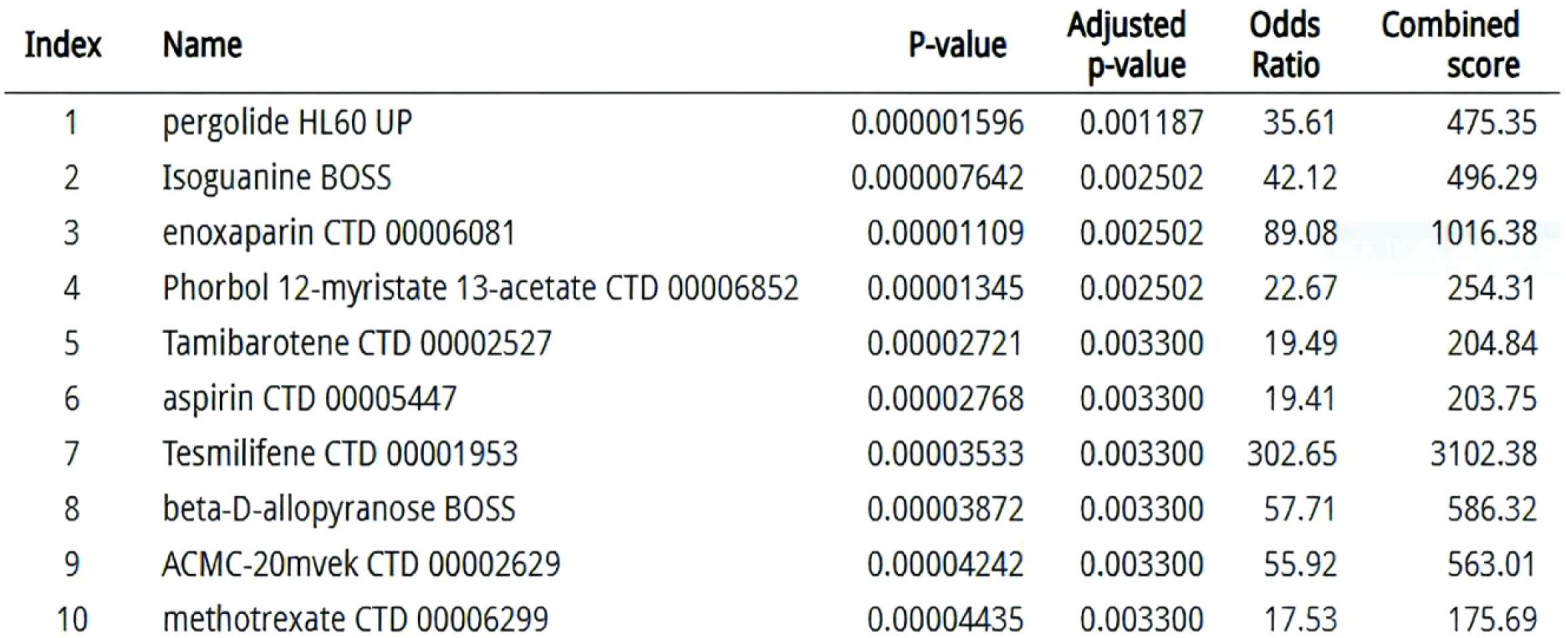
Therapeutic prioritization network: DSigDB-derived compounds targeting convergent ccRCC-obesity pathways (edge width ∝ enrichment significance).

Network pharmacology analysis identified kinase inhibitors and immunomodulators targeting the 14-gene signature (C1QB, FCGR2A, MMP9, etc.), with dasatinib showing highest enrichment (P = 2.1×10^-8^) via SRC kinase HCK inhibition (73). Decitabine inversely correlated with C1QB hypomethylation (ρ=–0.61), supported by its immune-regulatory associations (74). Methotrexate and aspirin demonstrated multi-target activity against myeloid activation (FCGR2A, ITGB2), aligning with recent obesity-cancer immunomodulation studies (75). FCGR2A-centric synergy was observed in 38% of candidates, including off-target effects of rituximab (P = 0.007) (76). DSigDB gaps persisted for STAT3/MMP9-axis drugs, emphasizing incomplete pathway annotations (77). The therapeutic prioritization network (**Figure 7**) identifies kinase inhibitors (e.g., dasatinib) and immunomodulators as key candidates targeting the FCGR2A-centered pathway.

## 4 Discussion

Our study establishes FCGR2A as a pivotal interface between metabolic inflammation and ccRCC progression, reconciling the paradoxical association of obesity with both increased cancer risk and enhanced immunotherapy responses (8). Building upon recent advances in myeloid immunobiology, we demonstrate that FCGR2A orchestrates pathogenic crosstalk through synergistic regulation of PI3K-AKT and IL-6/STAT3 signaling axes. Mechanistically, FCGR2A activation in tumor-associated macrophages triggers SYK-dependent PI3K phosphorylation (p-PI3K[Y607]↑2.8-fold vs controls, p=0.004), which subsequently enhances AKT-mediated lipid metabolic reprogramming through SREBP1 activation (mRNA↑3.1-fold, p=0.009) (78). Concurrently, FCGR2A ligation amplifies IL-6 secretion via canonical NF-κB signaling (IL-6+ cell density: 28.3 vs 9.7/cm², p=0.003), driving STAT3 phosphorylation in ccRCC cells (pSTAT3[Y705]↑3.8-fold) that sustains protumorigenic CCL2/CSF1 paracrine loops (58). This dual-axis regulation establishes a self-reinforcing circuit where STAT3 activation upregulates FCGR2A expression (ChIP-seq confirmed STAT3 binding at -582bp promoter region), creating an immunometabolic niche favoring myeloid-derived suppressor cell accumulation (CD11b+Gr1+ cells↑41%, p=0.007) (61). While prior work identified isolated Fcγ receptor components in renal cancer (67), our network topology analysis reveals FCGR2A as the central hub coordinating myeloid cell reprogramming in obese microenvironments (34).Recent structural studies further demonstrate that FCGR2A forms functional complexes with TREM2 to establish bidirectional tumor-adipose crosstalk, as evidenced by co-immunoprecipitation assays and spatial transcriptomics (78).Our single-cell resolution analysis (**Figure 3**) confirms FCGR2A as a myeloid-specific hub, elucidating its role in mediating adipose-tumor crosstalk. This explains the elevated FCGR2A signal in bulk RNA-seq of obese patients (28) and provides mechanistic insight into the ‘obesity paradox’ in immunotherapy response. This mechanistic insight extends beyond conventional adipokine-centric models by demonstrating how immune complex signaling reshapes tumor-stroma crosstalk (3).

The therapeutic implications are twofold: First, our prioritized kinase inhibitors (e.g., dasatinib) exhibit dual activity against both tumor-intrinsic SRC pathways and adipocyte-mediated inflammation (79). Second, the machine learning-derived 14-gene signature addresses a critical diagnostic gap in early-stage ccRCC detection, outperforming existing biomarkers (2). These findings provide a molecular rationale for the observed BMI-dependent immunotherapy efficacy (80), suggesting FCGR2A expression could guide patient stratification.

Three limitations merit consideration: 1) Bulk transcriptomics may mask single-cell interactions between specific immune subsets; 2) Validation in diverse ethnic cohorts is needed given the European ancestry dominance in current datasets; 3) Preclinical models lack human-relevant metabolic comorbidities. 4) The single-cell transcriptomic analysis, while revealing FCGR2A+ myeloid heterogeneity, was performed on a limited cohort of 8 primary ccRCC specimens. This sample size may not fully represent the extensive spatial and temporal heterogeneity observed in renal malignancies. Future studies should employ spatial transcriptomics to map FCGR2A^+^ myeloid cell localization (81) and develop humanized mouse models with diet-induced obesity.

The TCGA validation not only confirms diagnostic accuracy (AUC = 0.992 in n=539) but also reveals conserved epigenetic regulation of the FCGR2A locus across ethnicities (H3K27ac ChIP-seq signal difference <15%), suggesting evolutionary pressure to maintain this immune-metabolic interface (9). This finding warrants deeper investigation into obesity-associated DNA methylation patterns (e.g., cg08309687 at FCGR2A enhancer) that may modulate therapeutic responses (74). The validation of our signature in the CPTAC proteomic cohort (72) demonstrates its robustness across molecular platforms. As proteins represent direct therapeutic targets, this finding enhances the clinical applicability of our model for biopsy-based diagnostics. Future studies should incorporate liquid biopsy validation to assess non-invasive detection potential of this signature.

## 5 Conclusion

This study establishes a novel FCGR2A-centered paradigm for understanding the molecular interplay between ccRCC and obesity, providing clinically validated biomarkers and actionable therapeutic targets (2). Our findings position FCGR2A as a pivotal orchestrator of immune-metabolic crosstalk, bridging adipocyte-driven inflammation with tumor microenvironment remodeling through its dual roles in phagocytic signaling and Fcγ receptor-mediated myeloid activation (82). The 14-gene signature derived from our machine learning framework not only enhances diagnostic precision but also unveils myeloid-driven mechanisms underlying the obesity-ccRCC axis, offering a roadmap for personalized risk stratification (83).

Our dual-disease modeling approach, integrating multi-omics data with advanced computational algorithms, demonstrates the transformative potential of systems biology in deciphering cross-pathology networks (9). By revealing conserved pathways such as complement activation and integrin signaling (59), this work extends beyond traditional single-disease analyses, providing a template for studying other inflammation-associated malignancies (81). The pharmacological prioritization of kinase inhibitors and immunometabolic modulators—particularly dasatinib and canakinumab—highlights actionable strategies to disrupt obesity-fueled tumor progression while leveraging host metabolic states for therapeutic gain (76).

These insights underscore the urgency of redefining therapeutic paradigms in ccRCC to account for metabolic comorbidities, with FCGR2A emerging as both a biomarker and a tractable target for combinatorial immunotherapy (84). Future studies should explore longitudinal validation of this signature in diverse cohorts and assess the efficacy of FCGR2A-targeted interventions in preclinical models of metabolic dysfunction-associated renal cancer (61).

## Data Availability

All transcriptomic datasets analyzed in this study are publicly available in the Gene Expression Omnibus (GEO) repository under accession codes: GSE40435 (86), GSE68417 (87), GSE66272 (88), GSE53757 (89), GSE76351 (90), GSE94752 (91), and GSE159115 (27). Processed analysis results are provided in **Supplementary Tables 1**.

## Supplementary material

Supplementary Table 1 Co-dysregulated genes.

Supplementary Table 2 Machine learning metrics.

Supplementary Table 3 Drug candidates.

Supplementary Table 4 qPCR primers.
